# A novel method for cliff vegetation estimation based on the unmanned aerial vehicle 3D modeling

**DOI:** 10.3389/fpls.2022.1006795

**Published:** 2022-09-23

**Authors:** Minghui Li, Enping Yan, Hui Zhou, Jiaxing Zhu, Jiawei Jiang, Dengkui Mo

**Affiliations:** ^1^Key Laboratory of Forestry Remote Sensing Based Big Data and Ecological Security for Hunan Province, Changsha, China; ^2^Key Laboratory of State Forestry Administration on Forest Resources Management and Monitoring in Southern Area, Changsha, China; ^3^College of Forestry, Central South University of Forestry and Technology, Changsha, China; ^4^Guangxi Forest Inventory and Planning Institution, Nanning, China; ^5^Hunan Maoyuan Forestry Co., Ltd., Yueyang, China

**Keywords:** cliff, vegetation cover, structure from motion, unmanned aerial vehicle, close-range photogrammetry

## Abstract

The cliff ecosystem is one of the least human-disturbed ecosystems in nature, and its inaccessible and often extreme habitats are home to many ancient and unique plant species. Because of the harshness of cliff habitats, their high elevation, steepness of slopes, and inaccessibility to humans, surveying cliffs is incredibly challenging. Comprehensive and systematic information on cliff vegetation cover is not unavailable but obtaining such information on these cliffs is fundamentally important and of high priority for environmentalists. Traditional coverage survey methods—such as large-area normalized difference vegetation index (NDVI) statistics and small-area quadratic sampling surveys—are not suitable for cliffs that are close to vertical. This paper presents a semi-automatic systematic investigation and a three-dimensional reconstruction of karst cliffs for vegetation cover evaluation. High-resolution imagery with structure from motion (SFM) was captured by a smart unmanned aerial vehicle (UAV). Using approximately 13,000 records retrieved from high-resolution images of 16 cliffs in the karst region Guilin, China, 16 models of cliffs were reconstructed. The results show that this optimized UAV photogrammetry method greatly improves modeling efficiency and the vegetation cover from the bottom to the top of cliffs is high-low-high, and very few cliffs have high-low cover at the top. This study highlights the unique vegetation cover of karst cliffs, which warrants further research on the use of SFM to retrieve cliff vegetation cover at large and global scales.

## Introduction

A cliff is a unique geomorphic terrain that supports a diverse range of rare plants (Boggess et al., 2017). However, for humans, cliffs are hostile environments that make any explorations challenging. As an extremely crucial ecological environment, the relatively small size and fragility of habitats available for vegetation on cliffs make species extinction highly likely as cliff environments are very different from other environments. Because of its extremely hostile environments, systematic surveys of cliffs are challenging. Cliff information, such as vegetation coverage, biodiversity, and endemic species, has attracted much attention from many botanists, biologists, and environmental scholars (Fazio et al., 2019; Gerivani and Savari, 2020). Fractional vegetation cover (FVC) is defined as the projected percentage of the total study area that is vegetated (i.e., contains roots, stems, and leaves) (Wan et al., 2021). FVC is used to characterize the degree of vegetation cover; it is an important indicator of surface vegetation cover and the ecological environment. Therefore, vegetation coverage largely reflects the quality of the ecological environment of a cliff. The normalized difference vegetation index (NDVI) is used to detect both vegetation growth status and vegetation coverage, and to eliminate radiation errors. The band calculation of satellite images can be used as a direct reflection of vegetation coverage (Carlson and Ripley, 1997). Most vegetation cover research concentrated on variations of large-scale ground vegetation using the calculation of NDVI (Chen H. et al., 2021).

However, most cliffs have slopes of up to 90° and are small in scale, making it hard to calculate cliff vegetation cover using satellite images and traditional ground projection methods (Ferrer Velasco et al., 2022). In addition, because of the inaccessibility of cliffs (Zhou et al., 2021), classic large-scale vegetation surveys are not suitable for karstic cliff forest landscapes. Therefore, an accurate, efficient, and practical method for analyzing the vegetation cover of cliffs is urgently needed (Deng et al., 2022; Vitali, 2022). Calculation of vegetation cover in special and small-scale areas (such as sinkholes and cliffs) requires the use of aerial remote sensing and ground measurements such as terrestrial lidar and artificial climbing sampling (Boggess et al., 2021). So far, few studies have explored the vegetation coverage of cliffs, examples of which are the Tiankeng sinkhole (Jiang et al., 2021) and cliff flora (Strumia et al., 2020). The main difficulty of currently available cliff vegetation coverage measurements is that the cliffs are too high to allow for obtaining a manual control group, and it is difficult to efficiently conduct surveys on a large scale.

With the continuing development of technology, drones have become an indispensable tool for cliff surveys. Unmanned aerial vehicle (UAV) oblique photogrammetry technology uses a UAV with a camera to acquire data a certain survey area to be surveyed in one vertical direction and four inclined directions. The advantages of this method include a wide field of view, comprehensive data collection, and the ability to create high-precision digital elevation models (DEMs), digital surface models (DSMs), point clouds, and reconstructions (Huynh et al., 2021; Lastilla et al., 2021). However, three-dimensional (3D) model details are incomplete and still rely on other measurement methods such as laser radar (Yin et al., 2020; De Almeida et al., 2021) or manual patching of modeling details, which is time-consuming and costly. The traditional modeling method obtains a series of images of the target through real photography or online download, which are then imported into 3D software as reference drawings. The modeler manually recovers the target from the base 3D geometry based on personal experience. However, this approach has many limitations. Firstly, it requires a high level of modeler expertise. Secondly, in practice, the target to be reconstructed is all-embracing, and may be a leaf, a tree, or even a whole forest. These limitations obstruct the realization of high-quality 3D reconstruction by traditional manual modeling technology. Consequently, labor costs remain high and collecting relevant data remains time-consuming and labor-intensive. To meet the challenge of high-quality modeling (Burdziakowski, 2018), many state of the art in fields (Ali et al., 2019; Shafiq et al., 2022) use many technologies such as UAV and artificial intelligence for three-dimensional reconstruction.

Structure from motion (SFM) is currently widely used and has shown great potential in the field of high-efficiency and low-cost 3D reconstructions (Dering et al., 2019; Barrile et al., 2022). SFM is a technique for estimating 3D structures in a sequence of multiple two-dimensional images containing visual motion information. Firstly, a series of 2D images is sent to a computer and inter-matching of these images is used to infer camera parameters. Secondly, using the actual spatial coordinate system and the plane coordinate system of UAV images, perspective transformation is performed on UAV images. In addition, by using automatic computer graphics processing technology, aerial triangulation can be used to obtain spatial parameters. Thirdly, a network model is created using 3D point cloud data, which can restore the real scenery of the target body to the greatest extent possible.

SFM offers many powerful and effective techniques that help with terrain change monitoring (Cucchiaro et al., 2021). Several recent studies have highlighted that the use of SFM technology makes it possible to monitor geomorphology for many years in the same location (Hayakawa and Obanawa, 2020; Koukouvelas et al., 2020). Furthermore, SFM can also be applied to investigate coastal cliff stability, and historical UAV images can be analyzed to identify long-term geomorphic changes in retreating coastal dune areas (Mancini et al., 2017; Berquist et al., 2018; Grottoli et al., 2020). In related research on cliffs, scholars have used a combination of UAV photogrammetry and human identification to mark plants on the cliff surface and study the number of plant species as well as their distribution on the cliff (De Simone, 2020). In artificial intelligence, a rock block identification method was developed based on UAV photogrammetry (and its computer implementation) of cliff face rock (Wang et al., 2019). Recently, SFM has been used for the 3D reconstruction of coastal cliffs (Gonçalves et al., 2021).

Based on the advantages of SFM in these aspects and the characteristics of cliff research, this paper presents a semi-automatic UAV-based 3D vegetation cover measurement method. This method takes UAV surround photos of the cliff under unmanned interference conditions from multiple angles, reconstructs the 3D model of the cliff by SFM, and segments the point cloud to evaluate the cliff vegetation cover.

## Materials and methods

**FIGURE 1:**
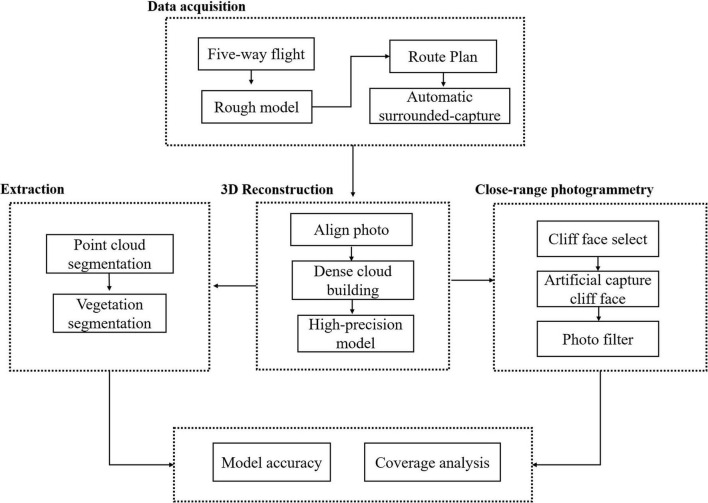
Flow of the cliff three-dimensional (3D) model reconstruction.

The following procedure was used to conduct the research (Figure 1). First, a rough 3D model was established by five-way flight, and route planning for the surrounding photography is carried out based on the rough 3D model. Then, the surrounding photos are obtained automatically by surrounding photography. Following this, an 3D reconstruction and a high-precision model are obtained. Both the vegetation point cloud and the non-vegetation point cloud are extracted by segmenting the dense point cloud. The cliff face is selected based on the high-precision model, and high-resolution cliff images are obtained through artificial close-range photogrammetry. Finally, the accuracy of the model and the vegetation coverage of the cliff are estimated by combining the point cloud extraction results and the high-precision cliff image set.

### Study area

In China, karst landscapes occupy a total of 130 km^2^, and the largest karst areas are in the provinces of Guangxi and Guizhou. In the mid-twentieth century, research on Chinese karst landscapes made significant progress. In the 1980s, research began to exploit the medicinal value, ecological restoration, and biodiversity of karst plants. The karst landscape of Guilin has undergone many alterations over the years, and today’s cliffs have autonomous summits with heavy flora cover at both the bottom and the top, as well as a badly weathered core region. Because of the extended lack of human disturbance, the escarpment’s biodiversity is higher than that of low-elevation plains.

**FIGURE 2:**
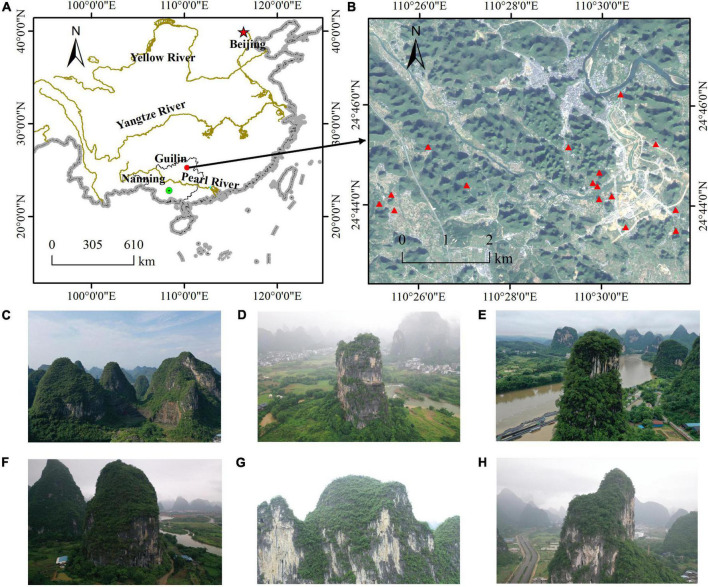
**(A)** The study site at Guilin (China). **(B)** The 16 cliffs in the study area. **(C–H)** The different sizes of cliffs.

The study area is located in a typical karst landscape area in Guilin, Guangxi Zhuang Autonomous Region, China (Figure 2A), contains 16 investigation points (Figure 2B). The surface morphology of the area is complex and diverse, and the landform type is mainly peaks depressions and peaks valleys, which mainly formed by the dissolution of carbonate rocks. Rock peaks are dense (Figure 2C), bedrock is exposed, showing complex and broken topography. It has received a lot of attention because of its unique topographic features (Figures 2D–H). The parent rock type is limestone and the soil type is red soil. This region has a subtropical monsoon climate with abundant sunshine throughout the year, rain and heat in the same period, mild climate, and average temperatures of 7.9°C in January, 28.0°C in July, and 18.8°C annually. Guilin is rich in vegetation types, with 70.91% forest coverage.

The process of selecting the study site has the following steps: firstly, the overall survey area is determined in the high-definition satellite images, and then after the field survey of the situation around the cliff, such as the independence of the cliff, whether it is a restricted area, etc., the cliff survey site is finally determined.

### Data acquisition and processing

Prior to images acquisition, typical cliffs covering the entire study area were pre-selected *via* Google satellite images as representatives for follow-up investigations. After on-the-spot investigation and flight tests, the 16 cliffs of Guilin were explored. The UAV DJI Mavic2 Pro was used to acquire the digital image datasets required for the 3D reconstruction of cliffs. The features of Mavic2 Pro are: 20 million effective pixels; 28 mm focal length; 31 min maximum flight time per battery; 907 g weight; compact and easy to carry; maximum flight altitude of 500 m; 1,080 p high-definition image transmission; maximum speed of 120 Mbps; the images taken can be viewed in real time; the import of self-designed routes is supported; low battery power automatically prompts automatic return.

**FIGURE 3:**
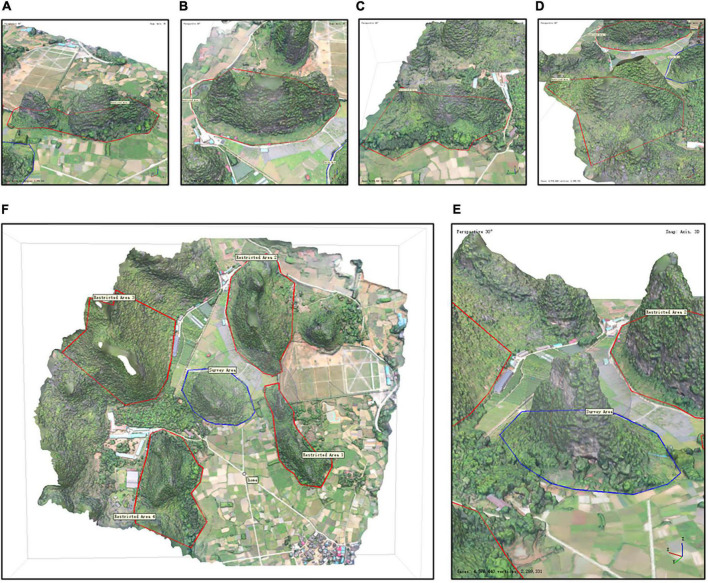
Survey area and restricted area of the study area. The area with the red edge is the restricted area, and the area with the blue edge is the survey area. **(A–D)** Orientation of the cliff: east, south, west, and north, respectively. There are five areas in **(F)**, including four restricted area and one survey area. **(E)** The area with the widest view in the study area.

However, the height of cliffs exceeds 100 m and the UAV must fly in the plane above the cliff, resulting in the unavailability of considerable information about the cliff, especially its bottom. Many studies have shown that in complex cliff conditions, the use of orthorectified and oblique images can reduce 3D modeling errors and optimize the survey route (Ali et al., 2019; Shafiq et al., 2022). The reason is that the more complex the angle of capture, the better the reproduction (Nesbit and Hugenholtz, 2019; Kozmus Trajkovski et al., 2020). Consequently, for this study, the five-way flight was utilized to build a rough 3D model, established a route based on the rough model for surrounded photogrammetry, and set up the restricted area and survey area (Figure 3).

**FIGURE 4:**
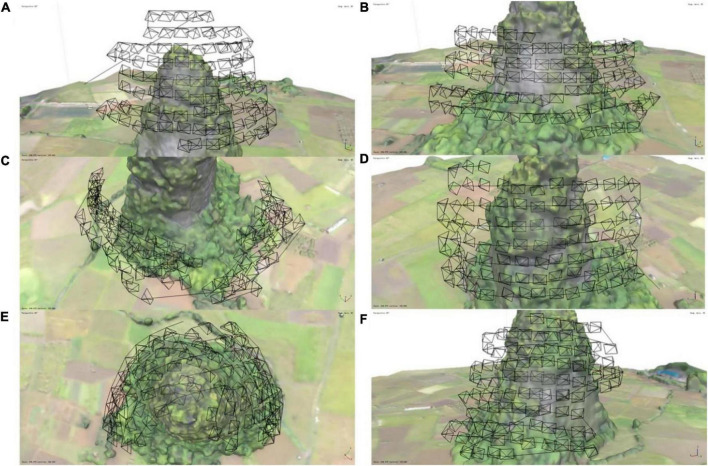
The survey route of the surrounding photography, showing 99 photograph points per flight route. **(A)** The first route, located at the top of the cliff. The first waypoint of **(B)** continues after the last waypoint of **(A)**, and so on. The last point of **(C–F)** completed photo capture, after which the drone returned automatically.

The rough 3D model information combined with the plan mission function of Agisoft Metashape 1.7.2 was used to automatically plan the route to the cliff in all directions based on the basic point cloud data (Figure 4). UAV survey parameters such as the minimum flight safety height, capture distance, and image overlap were set based on the actual size of the cliff. The vertical flight of drone will consume more electricity than the horizontal flight. Therefore, the route was designed so that the UAV moves horizontally before moving vertically to maximize the efficiency. The flight speed of the drone was 5 m/s. All routes ensured that every inch of the cliff could be photographed.

After the modeling is completed, close-range photogrammetry was also started from the bottom to top of the cliff, at a height interval of 10 m. Images of the cliff were taken as close as possible from a distance of 3 m from the cliff. After the modeling was completed, the close-range picture and 3D model were compared to locate the plant of cliff face for further vegetation cover evaluation.

### Cliff 3D reconstruction

A series of images obtained from the UAV survey was processed and the 3D reconstruction process was automated through Metashape. Dense point clouds and 3D mesh models were output. For the 3D reconstruction, several steps are necessary: (1) aligning photos; (2) reconstructing of dense point clouds; (3) denoizing the dense point clouds; (4) creating the 3D surfaces of the cliffs. The computer configuration and processing time of 3D reconstruction are shown in Table 1. After the 3D reconstruction was complete, the dense point cloud was segmented and divided into two categories: a vegetation point cloud and a non-vegetation point cloud. Then, the proportions of the two categories were calculated separately to evaluate vegetation coverage.

**TABLE 1:** Modeling hardware information.

GPU	CPU	Process time	Modeling precisions	Quantity
GeForce RTX 3060	Intel (R) Core (TM) i7-9700F	30 h	Medium	16

The processing time for all cliffs is 30 h, and the accuracy is set to medium (there is little difference in the practical application between medium and high accuracy settings; medium modeling accuracy was chosen because the processing speed for high modeling accuracy is slow, which affects the work progress).

The fineness and integrity of the 3D model were evaluated by judging whether the model structure is out of proportion, distorted, or deformed, and details are missing. The modeling data were analyzed from the following three aspects: (1) the relationship between the oblique photo and the surrounding photo; (2) the effect of the shooting distance on model accuracy; (3) the relationship between modeling photo and modeling time.

### Cliff vegetation cover estimation

Based on the 3D point cloud model for coverage calculation, there are two main steps: point cloud sampling and segmentation, followed by vegetation coverage calculation.

(1) Point cloud sampling

Noisy point clouds generated by natural conditions, human manipulation or machine errors greatly affect the experimental results. Therefore, the original 3D point cloud model must be denoized first, which is divided into ground point separation and vegetation denoizing (Chen K. et al., 2021).

(2) Segmentation of vegetation point clouds

The EXGI value is calculated using the RGB value of the point cloud, which is greater than 0.015 for vegetation and less than or equal to 0.015 for non-vegetation. Ground point clouds above 10 m were then filtered using high-pass filtering, and the cliff point clouds were segmented and divided into 10 gradients evenly ranging from low to high according to the elevation of cliff. The EXGI values for each gradient were used for comparative analysis of changes in plant cover.


(1) E⁢x⁢G⁢I=2⁢g-r-b

(2) R=*RRmaxG=*GGmaxB=*BBmax

(3) r=R*(R+*G+*B)*⁢g=G*(R+*G+*B)*⁢b=B(R+*G+*B)*

## Results

### Basic characteristics of cliff unmanned aerial vehicle data

By analyzing the cliff data, the number of photos, bottom perimeter, area, and volume of each cliff wall were obtained (Tables 2, 3). Analysis of these data and the practical survey uncovered an unusual rule: the modeling effect is not directly proportional to the modeling photos; the determinant of the modeling effect is the degree of plant growth and coverage. In general, the larger the height, volume, and area of the cliff, the more photos and shooting time are needed. Conversely, in practice, it was found that a small number of photos can be sufficient for building a satisfactory model for cliffs with larger rock area.

**TABLE 2:** Specifications of the produced unmanned aerial vehicle (UAV) images.

Number	Oblique photography	Surround photogrammetry	Relative height/m	UAV to cliff distance/m	Capture time/h
1	75	605	82	30	2.0
2	162	610	87	25	2.1
3	40	605	88	20	2.0
4	80	598	93	20	2.0
5	62	381	96	25	1.3
6	144	776	103	20	2.6
7	63	784	117	25	2.6
8	67	670	122	20	2.3
9	48	516	125	20	1.7
10	40	760	130	15	2.6
11	54	578	132	25	1.9
12	76	697	132	20	2.3
13	102	769	139	20	2.6
14	242	771	140	20	2.6
15	263	867	149	30	2.9
16	59	690	168	40	2.3

**TABLE 3:** The bottom perimeter, surface area, and volume of the cliff.

Numbers	Perimeter/m	Area/m^2^	Volume/m^3^		
			Above	Below	Total
1	366.6	9648.4	206257.3	1024.3	205233
2	291.4	5377.4	139565.1	3794.4	135770.7
3	364.0	9597.1	206274.3	695.968	205578.3
4	328.5	7969.5	235602.6	2123.1	233479.5
5	293.4	6319.6	135743.5	4020.6	131722.9
6	434.5	13906.7	547785.6	7676.4	540109.1
7	498.8	16797	502094.9	9535.2	492559.7
8	456.4	13859.1	441946.7	2821.2	439125.6
9	284.2	5835.4	241346	1941.9	239404.1
10	324.3	7117.8	407490.2	5580.8	401909.4
11	284.2	6222.9	303036.3	3587.7	299448.6
12	357.4	8991.5	371974.1	1366.2	370607.9
13	562.9	20949	719596.9	14471.1	705125.8
14	425.7	13559.3	705442.9	679.461	704763.4
15	611.5	26835.4	999571.7	548.928	999022.8
16	668.3	32744.9	2325530	6682.4	2318850

The plane of the cliff is higher than the flat bottom, so there will be a lower volume and an upper volume.

Table 2 presents basic information of the 3D modeling dataset. The results show that the number of oblique photographs is determined by the bottom orthophoto area and the vertical height of the cliff. The larger the bottom area, the higher the number of photos taken under the same flight conditions. In addition, the higher the height of the cliff wall, the larger the oblique photography area is to ensure the complete capture of details of the bottom of the cliff.

### 3D modeling results

**FIGURE 5:**
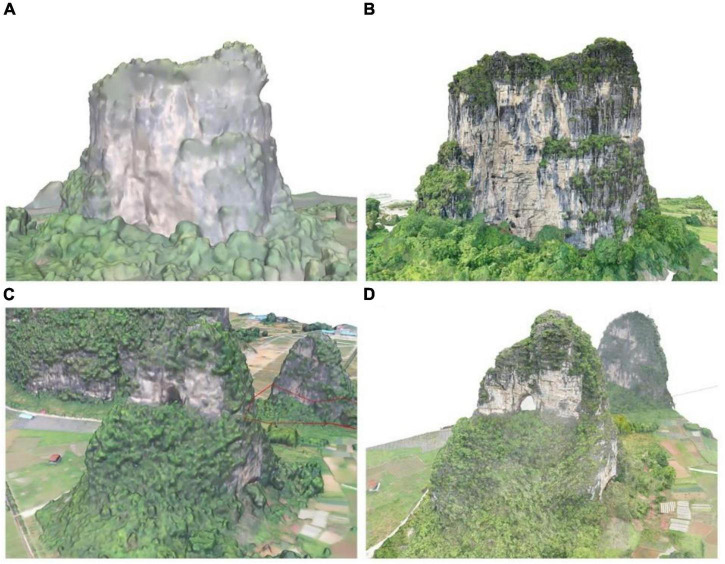
**(A)** Low-resolution model of No.13; **(B)** high-resolution model of No.13; **(C)** low-resolution model of No.14; **(D)** high-resolution model of No.14.

To test the feasibility of the design scheme, quality analysis was performed for texture details, the relationship between oblique photos and the effect of shooting distance on model accuracy. Further examination clearly showed, that the 3D model details are essentially without loopholes, have realistic and uniform tones, provide a complete and realistic display of the exterior information of the cliff, and present complete and clear texture details. Because the cliff 3D modeling used surrounding photography, the integrity of blind area identification and the high-resolution nature of the image data could be successfully increased. The comparison between the rough model and the high-precision model is shown in Figure 5. The texture of the model built by five-way flight is rough and lacks considerable texture details. The texture of the model built by flying around is clear, and can intuitively show the distribution of plants and the characteristics of the wall. Providing detailed models is very important for applications involving spatial analysis and realistic visualization.

By analyzing the relationship between data and model, the following two rules were covered:

(1) The relationship between oblique photos and surround photos
  The number of oblique images is not proportional to the surrounding photos. The significance of this lack of proportionality is that there is no need for excessively pursing a high number of photos taken by oblique photography. If sufficient overlap is ensured, it is still possible to set an automatic flight route for the collection of modeling data. As an example, there are 102 oblique photos and 769 surrounding photos of No.13 cliff, and 242 oblique photos and 771 surrounding photos of No.14 cliff. Compared with No.14 cliff, No.13 cliff has a large volume and a high height, and less photos were collected. The modeling results of No.13 cliff are better. The reason is that No.13 cliff has a high degree of exposure, and plants are mostly climbing plants and vines, with huge shrubs or trees only distributed on the top of cliff.
(2) The effect of shooting distance on model accuracy
  Research showed, that a shooting distance of 20 m is suitable, but the shooting distance is not the decisive factor for the accuracy of the model. Rather, the photo overlap determined by the shooting distance is the decisive factor. The farther the distance, the smaller the overlap, while the closer the distance, the greater the overlap.

### Cliff vegetation cover

**FIGURE 6:**
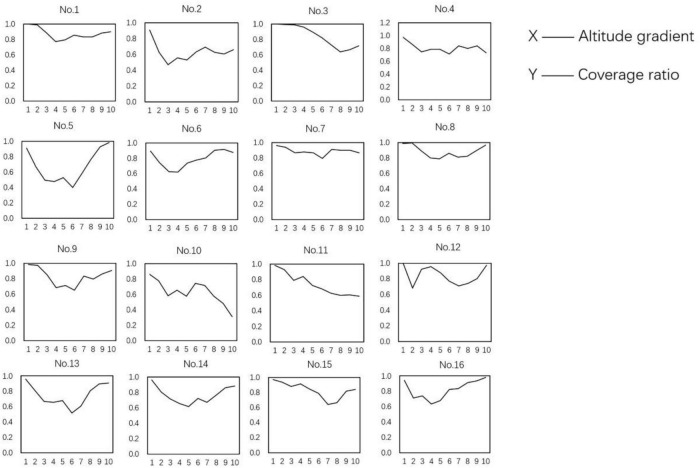
Gradient change of coverage. The vertical axis represents the relative height of the cliff divided into 10 gradients with equal distances within each gradient. The point cloud is divided into two categories after segmentation statistics *via* formulae (1), (2), and (3): plant point cloud and cliff point cloud. The *x*-axis shows the altitude gradient. The *y*-axis shows the ratio of the plant point cloud to the total point cloud.

Figure 6 shows that almost all cliffs have the highest coverage at both the bottom and top, with the lowest value occurring between gradients 3 and 7. From this, it can be predicted that the altitude will have the highest coverage within 30% of the overall altitude. If the vegetation at the top is sparse, rock exposure is considerable, and the cliff is highly unstable and sensitive to severe weathering. The chance of collapse is substantial. If there are residential area nearby, it can be monitored for a long time to predict collapse, especially on rainy days when the risk is greatest.

**FIGURE 7:**
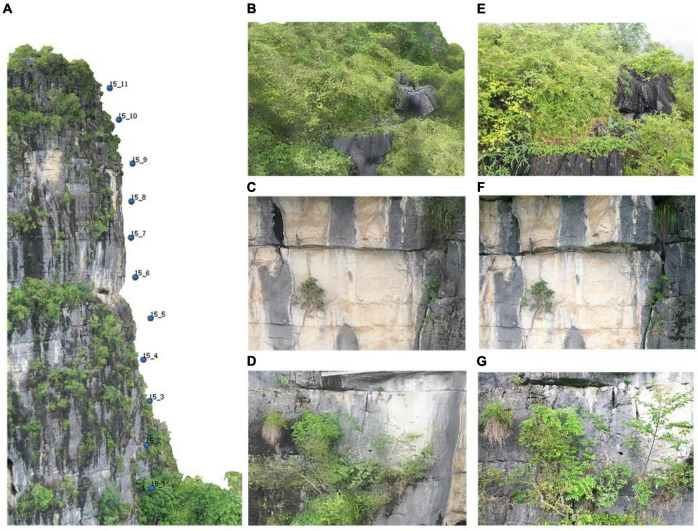
**(A)** Close-range photogrammetry of the cliff. In the picture, a total of 11 photos were taken of the cliff face, and the interval height of each photo was 10 m. Cliff number and shot order of close-range photo were renamed. **(B–D)** The upper, middle, and lower parts of the cliff model. **(E–G)** The upper, middle, and lower parts of close-range images.

Based on the high-precision model, high-resolution plant images were captured *via* close-range photogrammetry from the side of the cliff. The photo alignment function can match the high-resolution image with the model, so that the specific location of plants on the cliff can be quickly obtained. This is of great significance to the study of plant distribution. High security and long-term fixed-point monitoring can be realized. In Figure 7, the camera positions are distributed evenly on cliffs, and the model and drone photos are basically consistent.

## Discussion

### Model texture analysis

**FIGURE 8:**
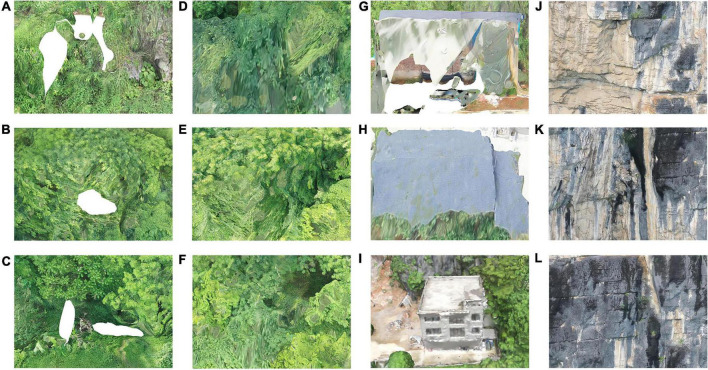
**(A–C)** Holes of vegetation in the lower part of cliff model. **(D–F)** Distortion of high vegetation cover on model surface. **(G–I)** Holes of house building in the lower part of cliff model. **(J–L)** Cliff rock without vegetation cover of the model.

There are a few holes on the surface of the cliffs and buildings as shown in Figure 8. Model holes and cracks in the 3D model can be repaired with software. Individual buildings are often distorted, deformed, and stretched as shown in Figure 8. Holes in roof models are generally cause by inadequate photo angles and heights. Buildings along or near hills without sufficient photo overlap are likely to show damage and deformation.

Analysis showed that in the cliff environment, vegetation is most prone to the phenomenon of fuzzy texture. Because of small changes in the position of rocks and buildings (Isokane et al., 2018), better results can often be achieved after re-shooting. Because of the high degree of the overlap, rocks on the cliffs cause a few problems such as drawing at the shelter of the eaves and fuzzy texture of the model details. Particularly the bottom of the cliff with more plants is prone to such problems. The solution is to take multiple shots, improve both shooting angle and overlap, and supplement the photo database. In the cliff environment, vegetation is the most prone to the phenomenon of distortion, while better results of rocks and buildings can be achieved after re-shooting because of long-term stability. In addition, we found that under the same flight conditions and modeling software, the UAV type had a slight effect on the modeling accuracy, but had little effect on the overall model accuracy.

### Factors influencing the modeling

In this paper, a UAV surrounded photogrammetry modeling method for karst cliff vegetation coverage is proposed. The method analyzes different features of the cliffs and automatically creates different 3D wrap-around UAV photography routes. These improved the integrity, quality, and efficiency of high-resolution UAV image acquisition of karst cliffs in extremely harsh conditions. The generated 3D model achieves the extraction of clear texture details, high authenticity, and reliable geometric accuracy. Further methods to improve the efficiency and accuracy of UAV flight using relevant methods will be considered in the future (Kose and Oktay, 2021). UAVs are now widely used to create high-resolution 3D models, particularly in the fields of engineering, surveying, and mapping. Image quality is crucial in UAV surveys which is directly influenced by the photographic angle, flight mode, and degree of image overlap. For photogrammetry, the distribution and number of ground control points (G) are important. In fact, no definite conclusion was found in the relationship between the distribution and number of GCPs and accuracy (Rock et al., 2011). However, modeling accuracy is primarily determined by the quality and quantity of photos taken, and GCPs have little impact. Moreover, GCPs can only be set in flat and open areas that are evenly distributed throughout the whole study area, which cannot be found in the steep terrain of these cliffs. Consequently, GCPs were not set in this study. Furthermore, because of the specificity of the cliff and the characteristics of vegetation modeling, the method used has limitations for modeling mountainous areas with high vegetation cover. Although a consumer-grade UAV can be used for estimating vegetation cover, airborne laser radar would achieve a more accurate representation. Issues such as how to estimate the parameters of the cliff to obtain more precise measurements should be thoroughly researched.

In addition, some photos, particularly in the area with plant abundance at the bottom of the cliff, cannot be photo aligned during SFM calculation, affecting the modeling effect. In this case, more photos were manually captured from the bottom of the cliff when planning the route. However, the disadvantage is that the terrain is greatly undulating, and the accuracy of the results obtained by covering and blocking of seriously undulating areas cannot meet the requirements. There are difficulties in data collection in no-fly areas and areas where satellite positioning cannot be performed. In this study, the JPG format was used for drone photos instead of the RAW format. The reason is that if the RAW format is used, the shooting time for each waypoint will be too long, which is time-consuming and seriously affects the work efficiency. Therefore, this is not conducive to modeling and processing of drone data.

### Acquisition of vegetation cover

Traditional measurement methods of vegetation coverage can be divided into ground measurement and remote sensing estimation methods. Ground measurements are often used at field scales, while remote sensing estimates are often used at regional scales. At present, many methods for measuring vegetation coverage using remote sensing have been developed. A more practical method is to use a vegetation index to approximate vegetation coverage. The most commonly used vegetation index is the NDVI. Vegetation coverage can be estimated by counting the pixel size of images captured by UAV remote sensing. The disadvantage is that the edge of a single photo will be distorted. Overall, currently used vegetation coverage surveys have in common that they require the surveyed area to be a flat surface. For example, areas covered by rocks such as sinkholes and cliffs will be ignored. Because of the limitations of the drone, the default photo format the drone uses during the collection process is JPG. The UAV images have red, green, and blue bands. Thus, the 3D model point clouds built using UAV images have RGB values and are suitable for calculating vegetation cover using the excess green index (EXGI). An in-depth literature screening shows that the EXGI contrasts the green portion of the spectrum against the red and blue to distinguish vegetation from the soil. This approach can also be used to predict NDVI and has been shown to outperform other indices that work with the RGB spectrum (Larrinaga and Brotons, 2019). Therefore, EXGI values were used to calculate vegetation cover in this study.

However, the method proposed here can overcome the limitations of traditional methods and vegetation coverage surveys can be conducted on 90° slopes. Although the presented vegetation coverage analysis method is fast and convenient, its disadvantage is that it is difficult to model high- vegetation coverage areas. Further research is needed to obtain a better solution for vegetation coverage calculation.

## Conclusion

The karst cliffs in Guilin are taken as experimental object, and a unique method for constructing a 3D model of cliffs was developed. This method uses the dense point cloud to calculate the EXGI value and the overall vegetation coverage. 3D modeling and coverage estimation of cliffs are difficult but nevertheless, very important. The proposed method can obtain the data required for such estimations and can do so in a relatively short time. This method allows the model to obtain a large amount of data for future research on cliff rock and changes in plant coverage. A historically constructed model can be used as a database to save information for future restoration of a specific cliff detail. The initial model was built based on a five-way flight to design the route around the flight, which saves a considerable amount of time compared with manual patching. Furthermore, this approach is beneficial for filling in the details of the cliff. In practice, it is much faster to model the details of the cliff by taking additional shots based on the original model. Further research will improve the algorithm to also analyze plant thickness, plant growth, and cliff stability in addition to vegetation coverage. As the modeling process imposes high requirements on photogrammetry software and computer hardware, it is necessary to automate and simplify various steps of the proposed method, which will be the direction of further research.

## Data availability statement

All data used in this study are available within the manuscript and any raw data can be obtained from the corresponding author upon reasonable request.

## Author contributions

EY and DM designed the study and provided comments. ML conducted the experiments and wrote the draft. DM improved the framework of the surveying method. HZ, JZ, and ML collected the field survey data. JJ and ML completed the calculations of cliff vegetation covers. EY and ML revised the manuscript. All authors read the manuscript, conceptualization, and methodology.
